# Nourin-Dependent miR-137 and miR-106b: Novel Biomarkers for Early Diagnosis of Myocardial Ischemia in Coronary Artery Disease Patients

**DOI:** 10.3390/diagnostics11040703

**Published:** 2021-04-14

**Authors:** Salwa A. Elgebaly, Robert H. Christenson, Hossam Kandil, Mohsen Ibrahim, Hussien Rizk, Nashwa El-Khazragy, Laila Rashed, Beshoy Yacoub, Heba Eldeeb, Mahmoud M. Ali, Donald L. Kreutzer

**Affiliations:** 1 Research & Development, Nour Heart, Inc., Vienna, VA 22180, USA; 2 Department of Surgery, UConn Health, School of Medicine, Farmington, CT 06032, USA; kreutzer@uchc.edu; 3 Department of Pathology, University of Maryland School of Medicine, Baltimore, MD 21201, USA; RCHRISTENSON@umm.edu; 4 Department of Cardiology, Kasr Alainy Faculty of Medicine, Cairo University, Cairo 11562, Egypt; Hossamkandil@kasralainy.edu.eg (H.K.); mibrahim_02@yahoo.com (M.I.); hussienrizk@gmail.com (H.R.); beshoy.ayuob@gmail.com (B.Y.); hebamostafakamel@gmail.com (H.E.); mahmoudbe1@hotmail.com (M.M.A.); 5 Department of Clinical Pathology-Hematology, Ain Shams Medical Research Institute (MASRI), Faculty of Medicine, Ain Shams University, Cairo 11566, Egypt; nashwaelkhazragy@med.asu.edu.eg; 6 Department of Biochemistry and Molecular Biology, Kasr Alainy Faculty of Medicine, Cairo University, Cairo 11562, Egypt; lailarashed@kasralainy.edu.eg; 7 Cell & Molecular Tissue Engineering, LLC, Farmington, CT 06032, USA

**Keywords:** Nourin, stable coronary artery disease, reversible myocardial ischemia, *miR-137*, *miR-106b-5p*, inflammatory biomarkers, ECHO Stress Test, ECG stress test, laboratory diagnostic test

## Abstract

Background: Although cardiovascular imaging techniques are widely used to diagnose myocardial ischemia in patients with suspected stable coronary artery disease (CAD), they have limitations related to lack of specificity, sensitivity and “late” diagnosis. Additionally, the absence of a simple laboratory test that can detect myocardial ischemia in CAD patients, has led to many patients being first diagnosed at the time of the development of myocardial infarction. Nourin is an early blood-based biomarker rapidly released within five minutes by “reversible” ischemic myocardium before progressing to necrosis. Recently, we demonstrated that the Nourin-dependent *miR-137* (marker of cell damage) and *miR-106b-5p* (marker of inflammation) can diagnose myocardial ischemia in patients with unstable angina (UA) and also stratify severity of ischemia, with higher expression in acute ST-segment elevation myocardial infarction (STEMI) patients compared to UA patients. Minimal baseline-gene expression levels of Nourin miRNAs were detected in healthy subjects. Objectives: To determine: (1) whether Nourin miRNAs are elevated in chest pain patients with myocardial ischemia suspected of CAD, who also underwent dobutamine stress echocardiography (DSE) or ECG/Treadmill stress test, and (2) whether the elevated levels of serum Nourin miRNAs correlate with results of ECHO/ECG stress test in diagnosing CAD patients. Methods: Serum gene expression levels of *miR-137*, *miR-106b-5p* and their corresponding molecular pathway network were measured blindly in 70 enrolled subjects using quantitative real time PCR (qPCR). Blood samples were collected from: (1) patients with chest pain suspected of myocardial ischemia (*n* = 38) both immediately “pre-stress test” and “post-stress test” 30 min. after test termination; (2) patients with acute STEMI (*n* = 16) functioned as our positive control; and (3) healthy volunteers (*n* = 16) who, also, exercised on ECG/Treadmill stress test for Nourin baseline-gene expression levels. Results: (1) strong correlation was observed between Nourin miRNAs serum expression levels and results obtained from ECHO/ECG stress test in diagnosing myocardial ischemia in CAD patients; (2) positive “post-stress test” patients with CAD diagnosis showed upregulation of *miR-137* by 572-fold and *miR-106b-5p* by 122-fold, when compared to negative “post-stress test” patients (*p* < 0.001); (3) similarly, positive “pre-stress test” CAD patients showed upregulation of *miR-137* by 1198-fold and *miR-106b-5p* by 114-fold, when compared to negative “pre-stress test” patients (*p* < 0.001); and (4) healthy subjects had minimal baseline-gene expressions of Nourin miRNAs. Conclusions: Nourin-dependent *miR-137* and *miR-106b-5p* are promising novel blood-based biomarkers for early diagnosis of myocardial ischemia in chest pain patients suspected of CAD in outpatient clinics. Early identification of CAD patients, while patients are in the stable state before progressing to infarction, is key to providing crucial diagnostic steps and therapy to limit adverse cardiac events, improve patients’ health outcome and save lives.

## 1. Introduction

The incidence of coronary artery disease (CAD) is very high among different populations worldwide, and approximately 15.5 million adult persons >20 years have CAD [1,2]. Because of lack of a simple laboratory test that can early detect myocardial ischemia in CAD patients, many of them are being first diagnosed at the time of the development of myocardial infarction. Currently, various cardiovascular imaging techniques including dobutamine stress echocardiography (DSE) [3], exercise ECG, CT coronary angiography, and coronary angiography are the cornerstone in the diagnosis of myocardial ischemia in intermediate and high-risk groups [4,5]. However, these tests have variable sensitivity and specificity across different studies [6] and each of these tests has its own limitations [7]. Since there no blood-based biomarker exists that can diagnose myocardial ischemia in CAD patients before progressing to infarction, developing a simple laboratory test for identification of myocardial ischemia in individuals who have CAD in the stable state is key to provide crucial diagnostic steps and early therapy to limit adverse cardiac events [2].

Pathological changes during myocardial ischemia are initiated within 10 min after interruption of coronary blood-flow and the myocardium ceases its aerobic metabolism [8]. Persistence of ischemia triggers a cascade of events, starting with a decreased diastolic and later systolic function, appearance of wall motion abnormalities and finally ECG abnormalities and development of angina [9,10]. Although cardiac biomarkers such as Troponin I and T are the gold standard diagnostic biomarkers for myocardial infarction [11,12,13,14,15], challenges related to sensitivity and specificity, as well as ability to rule-out reversible ischemia, may limit their use as biomarkers for the diagnosis of CAD patients [12]. Therefore, there is a clear need to identify and validate new blood-based biomarkers to identify “reversible” ischemic injury in stable CAD patients before progressing to infarction.

Elgebaly et al. [16,17,18,19,20,21,22,23,24,25] reported that Nourin® is a 3 KDa formyl peptide released within five minutes of the onset of ischemia by human and animal hearts in response to ischemic injury. Nourin is unique because of its rapid release by “reversible” ischemic myocardium when cells are “injured”, but still “viable” and “not dead”. If ischemia persists, Nourin is also released by necrotic cells [16,17,18,19,20,21,22,23,24,25]. Nourin is a potent inflammatory mediator and its release is associated with post-ischemic cardiac inflammation in early ischemia/reperfusion animal models of AMI, cardiopulmonary bypass surgery and heart failure [16,17,24]. Nourin was referred to as a “cardiac-derived leukocyte chemotactic factor” and was purified from cardioplegic solutions collected during cardiac arrest (“reversible” myocardial ischemia) from patients who underwent cardiopulmonary bypass surgery for coronary revascularization, and its amino acid sequence was also determined [17,18].

Using Nourin antibody ELISA and chemotaxis assay, high levels of Nourin were detected in blood samples collected at presentation to hospital Emergency Departments (EDs) from symptomatic acute coronary symptoms (ACS) patients and patients with documented AMI within the first eight hours of chest pain. Nourin was not elevated in symptomatic non-cardiac patients and healthy subjects [25]. These results suggest the potential use of the Nourin biomarker to “rule-in” myocardial ischemia in ACS patients and “rule-out” cardiac injury for symptomatic patients having non-cardiac causes. Additionally, using the Nourin amino acid sequence, an integrated Bioinformatics analysis was recently conducted and indicated that Nourin is a direct target for *miR-137* (marker of cell damage) and *miR-106b-5p* (marker of inflammation) in myocardial ischemic injury [21,22,23]. Using quantitative real time PCR (qPCR) results indicated that the Nourin-dependent *miR-137* and *miR-106b-5p* can diagnose myocardial ischemia in patients with UA and also stratify severity of ischemia, with higher expression in acute STEMI patients compared to UA patients. Minimal baseline-gene expression levels of Nourin miRNAs were detected in healthy subjects [21,22,23].

**Figure 1:**
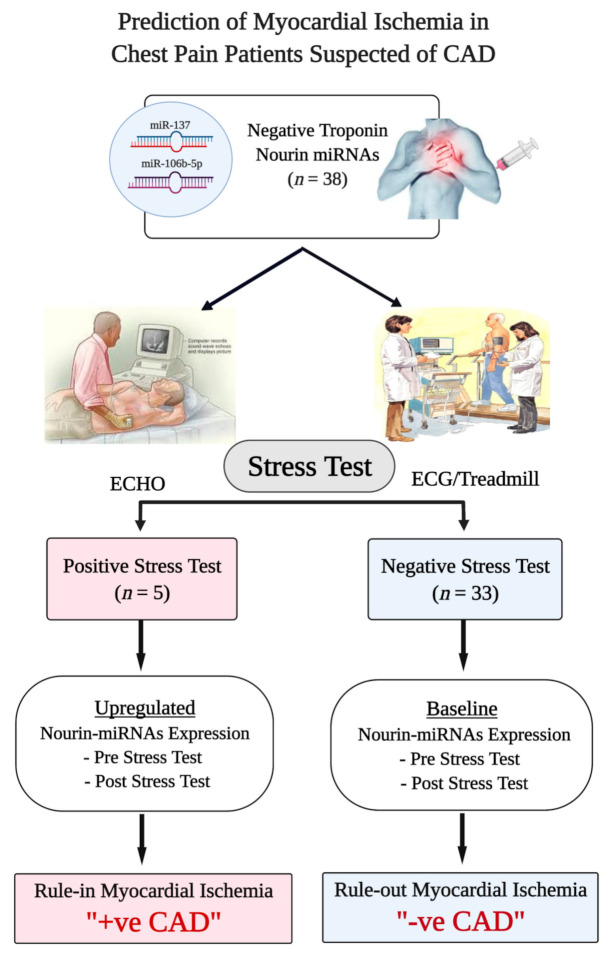
Flowchart Study Design. Abbreviations: CAD: coronary artery disease, ECHO: echocardiography, ECG: electrocardiogram.

In the present study, we tested the hypothesis that the Nourin-dependent *miR-137* and *miR-106b-5p* will be elevated in chest pain patients with myocardial ischemia suspected of CAD, who also underwent a DSE or ECG stress test, and that the elevated levels of Nourin miRNAs correlate with results of ECHO/ECG stress test in diagnosing CAD patients. As indicated in Figure 1, all chest pain patients suspected of myocardial ischemia (*n* = 38) were tested for Troponin. Patients with negative Troponin (levels below the clinical decision—99th percentile), underwent ECHO or ECG stress test. Patients with positive stress test also had significant upregulation of Nourin miRNAs both “pre-stress test” and “post-stress test”, while patients with negative stress test showed baseline values of Nourin miRNAs, also “pre-stress test” and “post-stress test”. Since results of this preliminary study indicate a correlation of Nourin miRNAs serum expression levels with ECHO/ECG stress test findings in determining myocardial ischemia and diagnosing CAD patients both “post-stress test” and “pre-stress test”, this suggests a promising role for the novel Nourin biomarkers to “rule-in” suspected CAD patients in “outpatient clinics”.

## 2. Results

### 2.1. Demographic Characteristics of Participants

The demographic and clinical characteristics of participants are presented in Table 1. Chest pain patients suspected of CAD who underwent ECHO/ECG stress test group (*n* = 38) represent the main core group of the study. Two additional control groups were enrolled: acute STEMI patients (positive control) and healthy subjects (negative control). All subjects enrolled in the study had met the inclusion/exclusion criteria and showed normal renal function (serum creatinine level < 1.2 mg/dL). As indicated below in Table 1 and Table 2, five out of the 38 chest pain patients showed positive ECHO/ECG stress test, while the rest of the 33 patients showed negative stress test. Because of reagent limitations, serum samples of only seven patients out of the 33 negative stress test patients were used to determine the levels of Nourin molecular network.

### 2.2. Nourin Network Gene Expression Profiles in Patients Suspected of Myocardial Ischemia

Chest pain patients suspected of myocardial ischemia (*n* = 38) underwent standard stress tests using dobutamine stress echocardiography (DSE) or exercise ECG/Treadmill stress test for the diagnosis of stable CAD. The Nourin-dependent miRNAs and their related molecular network genes were measured blindly in serum samples collected immediately “pre” the DSE or exercise ECG stress test, as well as 30 min “post” termination of the test from symptomatic patients suspected of myocardial ischemia. As indicated below, five out of the 38 chest pain patients showed positive ECHO/ECG stress test, while the rest of the 33 patients showed a negative stress test. Because of reagent limitations, serum samples of only seven patients out of the 33 negative stress tests patients, were used to determine the levels of Nourin molecular network.

#### 2.2.1. Assessment of *CTB89H12.4*/*miR-137*/*FTHL-17* Network “Post-Stress Test”

**Figure 2:**
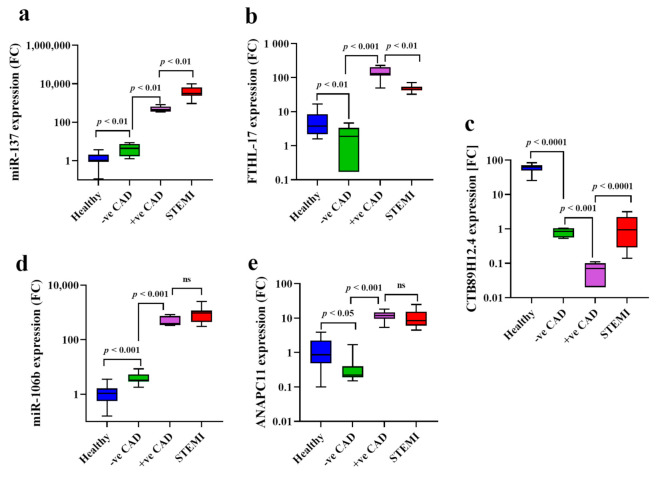
Boxplot graphs demonstrating the serum expression levels of *miR-137* in patients with chest pain and suspected for CAD; higher expression levels of *miR-137* (**a**), *FTHL-17* (**b**), *miR-106b* (**d**) and *ANAPC11* (**e**) were significantly associated with CAD; *lnc-CTB89H12.4* was downregulated (**c**); Abbreviations: CAD: coronary artery disease, STEMI: ST-segment elevation myocardial infarction, *FTHL-17*: Ferritin heavy chain like 17, *ANAPC11*: anaphase promoting complex subunit, *p* < 0.05 considered significant, ns: no-significant difference.

The expression levels of Nourin-dependent genes were measured blindly in serum samples of outpatients with chest pain suspected of myocardial ischemia using qPCR. The assessment was performed 30 min after exercise-induced ischemia (stress test). Upregulation of *miR-137* and *FTHL-17*, which are significantly associated with induced ischemia, are reflected by high expression levels post-stress test as presented in Table 2 and Figure 2. Specifically, Nourin-dependent *miR-137* and *FTHL-17* gene expression levels were significantly correlated with results obtained from ECHO or exercise ECG stress tests in determining the presence or absence of myocardial ischemia. As indicated in Figure 2, *miR-137* (Figure 2a) was upregulated by 572-fold and 69-fold for *FTHL-17* (Figure 2b) in serum expression of patients with positive stress test compared to negative stress test patients (*p* = 0.001), results which are consistent with the positive ECHO and ECG findings. Furthermore, when gene expression levels were compared in negative stress test patients to healthy controls, results showed higher expression levels of *miR-137* (4.2-fold, *p* = 0.01) (Figure 2a) in negative stress test compared to healthy, while for *FTHL-17*, there was higher gene expressions (2-fold, *p* = 0.01) (Figure 2b) in healthy subjects compared to negative stress test patients. Additionally, when gene expression levels were compared between acute STEMI patients and patients with positive stress test, a statistically significant difference was detected for the expression of *miR-137* (1.22-fold, *p* = 0.01) (Figure 2a), while *FTHL-17* was reduced by 2.6-fold in acute STEMI patients compared to positive stress test (*p* = 0.01) (Figure 2b). The median expression level of the candidate genes and the fold changes are presented in Table 2.

#### 2.2.2. Assessment of *CTB89H12.4/miR-106b-5p/ ANAPC11* Network” Post-Stress Test”

The serum expression levels of Nourin-dependent *miR-106b-5p* and *ANAPC11* were measured blindly in serum samples of patients with chest pain suspected of myocardial ischemia. The assessment was performed 30 min after exercise-induced ischemia (stress test). There was an upregulation of *miR-106b-5p* and *ANAPC11* post-stress test which are significantly correlated with results obtained from ECHO or exercise ECG stress tests in determining the presence or absence of myocardial ischemia. As indicated in Table 2 and Figure 2, serum levels of *miR-106b-5p* (Figure 2d) and *ANAPC11* (Figure 2e) were significantly increased by 122-fold and 60-fold, respectively, in patients with positive stress test (*p* = 0.0001) compared to negative stress test patients, results which are consistent with the ECHO and ECG findings. In addition, higher *miR-106b-5p* (Figure 2d) (three-fold increase, *p* = 0.001) was detected in patients with negative stress test compared to healthy controls (*p* < 0.01), while for *ANAPC11* (Figure 2e), there were higher gene expressions (4.5-fold, *p* = 0.04) (Figure 2e) in healthy subjects compared to negative stress test patients. Furthermore, when gene expression levels were compared between acute STEMI patients and patients with positive stress test, there was no statistically significant difference for gene expression of *miR-106b-5p* (1.8-fold increase, *p* = 0.06) and *ANAPC11* (1.4-fold increase, *p* = 0.06) (Figure 2d). The median expression level of the candidate genes and the fold changes are presented in Table 2.

Gene expression level of *lnc-CTB89H12.4* tends to be downregulated in ischemic injury. Contrary to the upregulation of *miR-137*, *FTHL-17*, *miR-106b-5p* and *ANAPC11,* a significant reduction level of *lnc-CTB89H12.4* by 11.4-fold was detected in serum of patients with chest pain who were positive to stress test compared to negative stress test patients (*p* < 0.01) (Figure 2c). Similarly, a significant difference was detected between patients with negative stress test and healthy subjects (*p* = 0.0001), and between STEMI patients compared to patients with positive stress test (*p* = 0.0001) (Figure 2c). Additionally, serum l*nc-CTB89H12.4* expression was decreased by 81-fold in patients with negative stress test compared to healthy controls (Figure 2c), and by 12.8-fold in STEMI patients compared to patients with positive stress test (*p* < 0.0001) (Figure 2c), respectively. The median expression levels of genes are presented in Table 2. 

### 2.3. Assessment of Nourin-Dependent miR-137 and miR-106b Gene Expression Profiles “Pre-Stress Test” and “Post-Stress Test” (30 Minutes After Termination) in Chest Pain Patients Suspected of Myocardial Ischemia 

**Figure 3:**
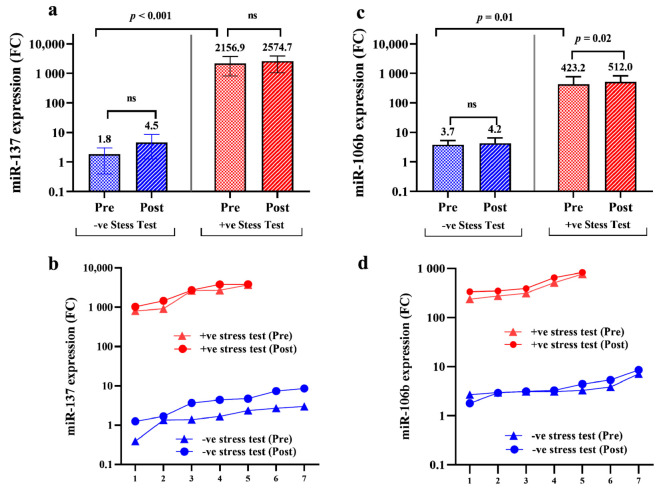
Bar chart graph illustrates *miR-137* (**a**) and *miR-106b-5p* (**b**) expression levels in patients with chest pain and suspected of CAD; miRNA expression levels were measured before stress test-induced ischemia, “pre-stress test” and 30 min “post-stress test”; the “expression patterns” of Nourin miRNAs, *miR-137* (**c**) and *miR-106b-5p* (**d**) were plotted for positive stress test patients “pre-stress test” and 30 min “post-stress test” and compared to patients who showed negative results in order to investigate the computability of Nourin miRNAs level and stress test results; *p* < 0.05 considered significant; ns: no-significant difference.

Gene expression profiles of *miR-137* and *miR-106b-5p* were compared in “pre-stress test” and “post-stress test” samples of positive and negative stress test patients in order to: (1) determine whether Nourin miRNAs are detected in blood circulation before stress-induced ischemia, and (2) evaluate the potential clinical use of Nourin miRNAs in diagnosing chest pain patients suspected of myocardial injury in outpatient clinics. Results indicated marked upregulation by 1198-fold (median: 2157) in *miR-137* expression level in “pre-stress test” patients with positive stress test compared to negative stress test patients (median: 1.8) (*p* < 0.001) (Figure 3a). Similar results were obtained for *miR-106b-5p* with upregulation by 114-fold (median: 423.2) in “pre-stress test” patients with positive stress test compared to negative stress test patients (median: 3.7) (*p* = 0.01) (Figure 3c). Furthermore, gene expression patterns for Nourin *miR-137* (Figure 3b) indicated no statistical significance was reached (*p* > 0.05) between “pre-stress test” and “post-stress test” samples in patients with positive and negative stress test. However, there was a slight elevation of the inflammatory marker, *miR-106b-5p* (Figure 3c) in patients with positive stress test (*p* = 0.02) detected “post-stress test” (median: 512.0) compared to “pre-stress test” (median: 423.2), which may likely reflect an ultimate association between *miR-106b-5p* and inflammatory cytokines associated with ischemia-induced aggravation and tissue hypoxia. 

## 3. Discussion

Despite the progress made in developing diagnostic tests to identify patients with stable CAD, there are still challenges and obstacles facing clinicians in diagnosis for this patient population without resorting to invasive interventions [2]. Currently, diagnosis of stable CAD depends on detecting ischemia by different stress test modalities or by detecting significant coronary artery stenosis using CT coronary angiography. However, these tests have variable sensitivity and specificity across different studies and some limitations such as radiation exposure and contrast use in CT coronary angiography [26], or suboptimal echocardiographic window in dobutamine stress echocardiography [27,28]. One approach to overcome these limitations is the use of blood-based biomarkers of myocardial ischemia for early diagnosis of CAD.

Recently, many efforts have been focused on exploring the development of early, simple, sensitive and non-invasive diagnostic blood-based biomarkers for CAD in order to provide crucial therapy to reduce the progression of the disease and lower the mortality rate [29]. However, measuring biomarkers in individuals with suspected CAD who underwent a stress test showed variable results across different studies. Highly-sensitive cardiac Troponin (hs-cTn) is one of the most commonly studied biomarkers to diagnose CAD, but Troponin level before stress test had low sensitivity in diagnosing CAD [30]. Wongpraparut et al. [31] reported that elevated Troponin level had 43% sensitivity in predicting moderate to severe ischemia by pharmacological stress test. Another study by Suchlez et al. [32] reported that elevated Troponin level had 53% sensitivity in detecting myocardial ischemia in patients with significant coronary artery stenosis diagnosed by coronary angiography. Meanwhile, Braunwald et al. [33] indicated that there is a quantifiable rise in Troponin level in patients who developed myocardial ischemia during exercise stress test and that the magnitude of this rise was proportional to the degree of ischemia on perfusion imaging. However, other studies demonstrated that inducible ischemia did not change Troponin level 18 min. or 4 h after the end of stress test [34]. Furthermore, since the rising pattern of Troponin after stress test is variable with inconsistent results in different studies [35], it represents a limitation that affects the diagnostic and prognostic value of Troponin for CAD patients [32].

MicroRNAs (miRNAs) have been illustrated to regulate different cellular biological processes and regulate gene expression, therefore they are superior to protein for early diagnosis of inflammatory and cardiac diseases. Recently, circulating miRNAs have been established as new markers of disease, including cardiac injury and inflammation [13,21,22,23,29,36,37,38]. Previous studies have demonstrated the diagnostic potential of exosomal miRNAs such as *miR-149-5p*, *miR-942-5p*, *miR-133b*, *miR-21* and *miR-32-5p* in stable CAD patients [39,40]. Additionally, a panel of circulating microRNAs were found to serve as early diagnostic biomarkers for stable CAD [41], whereas higher expression levels of *miR-765*, *miR-483*, *miR-122* and *miR-155* were demonstrated early, but *miR-149* and *miR-451a* were downregulated [42] in these stable CAD patients.

Using the Nourin amino acid sequence, we conducted an integrated bioinformatics analysis which indicated that Nourin molecular network is composed of *lncRNA-CTB89H12.4, miR-137, miR-106b-5p*, *mRNA-FTHL-17*, and *mRNA-ANAPC11* and that they are an autophagy-related RNA-based network linked to each other and to cardiovascular ischemia. Recently, Elgebaly et al. [21,22,23] reported that the Nourin-dependent *miR-137* was up-regulated by 1185-fold in unstable angina patients compared to healthy volunteers, and by 2.5-fold in acute STEMI patients compared to UA patients. Similarly, the Nourin-dependent *miR-106b-5p* was up-regulated by 150-fold in UA compared to healthy volunteers and by 4.6-fold in acute STEMI compared to UA patients. Unstable angina patients were confirmed by invasive coronary angiography, where all patients showed stenosis greater than 50% and Troponin level below the clinical decision limit. Baseline values of *miR-137* and *miR-106b-5p* were detected in healthy subjects. Furthermore, receiver operator characteristics (ROC) analysis revealed that the two miRNAs were sensitive and specific biomarkers for assessment of UA and STEMI patients [21]. We also reported that *miR-137* and *miR-106b-5p* were significantly correlated to *FTHL-17* and *ANAPC11*, respectively, while *lnc-CTB89H12.4* was negatively correlated to Nourin-dependent *miR-137* and *miR-106b-5p* [21,22,23]. Elgebaly et al. [24] also, reported that Nourin miRNAs were significantly upregulated in the standard Isoproterenol (ISO) rat model of heart failure (HF) induced by demand ischemia. Gene expression profiles showed upregulation of *miR-137* and *miR-106b-5p* by 8.6-fold and 8.7-fold, respectively, in the heart failure of ISO/saline rats compared to control/saline group. Interestingly, treating ISO “HF rats” with our bioenergetic drug, Cyclocreatine Phosphate (CCrP) prevented ischemic injury and the development of heart failure, resulting in normal ejection fraction and physical activity [16,19]. CCrP treatment also markedly reduced gene expression of miR-137 by 75% and of *miR-106b-5p* by 44% in the ISO/CCrP rats, “non-HF rats,” compared to ISO/Saline rats, “HF rats” [24]. Consistent with our results, *miR-137* was reported by others to be linked to ischemic injury, where *miR-137* exacerbates apoptosis of ischemia-reperfusion injured cardiomyocyte [43]. Recently, *miR-106b-5p* was reported to be implicated in pathogenesis of myocardial ischemic injury, where higher expression level of *miR-106b-5p* was demonstrated in cardiomyocyte cells immediately after exposure to hypoxia and apoptosis [44].

In the current study, we evaluated: (1) whether the Nourin-dependent miRNA network can detect myocardial ischemia in chest pain patients suspected of CAD, and (2) whether the elevated levels of Nourin miRNAs in CAD patients correlate with the positive diagnosis of myocardial ischemia by dobutamine stress echocardiography or exercise ECG tests. Additionally, we compared gene expressions of Nourin-dependent miRNAs in stable CAD patients to levels detected in acute STEMI patients (Nourin positive control) and healthy subjects (Nourin negative control). A total of 70 subjects who met the inclusion/exclusion criteria were enrolled in this study. Thirty-eight (*n* = 38) consecutive chest pain patients, in whom CAD was suspected, underwent DSE or ECG stress test. “Pre-stress test” and “post-stress test” of circulating *miR-137* and *miR-106b-5p* expression levels were tested blindly in patient’s sera using quantitative real time PCR (qPCR). Additionally, miRNAs targets genes (*FTHL-17* and *ANAPC11*) and lncRNA regulatory gene (*CTB89H112.4*) were tested in parallel to the miRNAs. Results indicated that a strong correlation was observed between Nourin miRNAs serum expression levels and results from ECHO/ECG stress test in diagnosing myocardial ischemia in CAD patients. There was a significant elevation of Nourin-dependent *miR-137* and *miR-106b-5p* in patients with inducible myocardial ischemia by stress test (positive CAD) compared to symptomatic patients with no inducible myocardial ischemia (negative CAD). Specifically, “post-stress test” positive CAD patients showed upregulation of *miR-137* by 572-fold increase and *miR-106b-5p* by 122-fold when compared to negative test patients (*p* < 0.001). Similarly, “pre-stress test” showed upregulation of *miR-137* by 1198-fold and *miR-106b-5p* by 114-fold in comparison to negative test patients (*p* < 0.001). 

The observed high miRNA level “pre-stress test” can possibly be explained by the continuous steady-state release of Nourin into the circulation in response to the chronic myocardial ischemia state in CAD patients. It is likely that the small size of Nourin (3 kDa) facilitates its transport across cell membranes from viable ischemic cardiomyocytes leading to its continuous presence in blood circulation before induction of ischemia. Although CAD patients continued to show elevation of Nourin miRNAs after inducible myocardial ischemia, there was no significant difference in the elevated levels of *miR-137,* as a marker of “cell damage” both “pre-stress test” and “post-stress test” (*p* < 0.275). However, there was a slightly higher levels of the “inflammatory” marker, *miR-106b-5p,* “post-stress test” compared to “pre-stress test” (*p* = 0.02), suggesting a role of ischemia-induced inflammation. Healthy subjects’ sera collected “post-stress test” showed minimal baseline-gene expression levels of Nourin miRNAs. 

It has been reported that the prevalence of myocardial ischemia in patients with intermediate pre-test probability for obstructive CAD is only 14% [45,46]. Although in our study the small sample size of CAD patients (*n* = 5) is a limitation, it is attributed to the small percentage of patients in whom the stress test turned to be positive for myocardial ischemia among the total number of patients (*n* = 38). The 5 CAD patients constitute 13%, which is comparable to published reports [45,46], while for the remaining 33 negative patients, for four of the test was inconclusive and 29 had normal stress test. Larger clinical studies are needed to determine the potential clinical utility of the Nourin gene-based test in identifying myocardial ischemia in CAD patients.

## 4. Subjects & Methods

### 4.1. Bioinformatics Analysis

Bioinformatics analysis was conducted to identify the differentially expressed genes (DEGs) that are related to Nourin amino acid sequence and also linked to myocardial ischemic injury, as we previously reported [21]. Briefly, three genes’ expression profiles were downloaded from the Gene Expression Omnibus (GEO) database and integrated Bioinformatics analysis was applied. Initially, the functional gathering analysis was used to map the Nourin protein-genes interaction using the Kyoto Encyclopedia of Genes and Genomes (KEGG), as well as incorporated gene ontology (GO) databases. In order to explore the biological characteristics of the DEGs, an interaction network of protein–protein internet (PPI) was created using the STRING database. Two upregulated genes named *“Ferritin heavy chain like 17 (FTHL-17) and Anaphase Promoting Complex Subunit (ANAPC11)”* were selected based on the following: (1) direct targets of Nourin protein, (2) expressed on normal myocardium tissue, and (3) significantly dysregulated in ischemic injury. Subsequently, we constructed the *lncRNA/miRNA/mRNA* networks to predict the up-stream regulating genes of Nourin. Using the *miRDB*, *TargetScan* and *miRTarBase* databases, two *miRNAs* “*miR-137* and *miR-106b*” were found to regulate the expression of *FTHL-17* and *ANAPC11* genes, respectively. Furthermore, *Starbase* and *CHIP*, as well as *DIANA-LncBasev3* databases were used to predict the *lncRNA*. The *lncRNA* “*CTB89H12.4*” was found to regulate the expression of both *miR-137* and *miR-106b*. To ensure accuracy of the obtained data, we used three different databases to identify the high complementarity binding sites between different genes and Nourin protein. Finally, each network “[*FTHL-17/miR-137/lnc-CTB89H12.4*] and [*ANAPC11/miR-106b/lnc-CTB89H12.4*]” was verified using the Clustal 2 multiple sequence alignment software for the validation of the gene–gene alignment. 

### 4.2. Study Design and Protocol

#### 4.2.1. Study Participants

A total of 70 subjects who met the inclusion/exclusion criteria were enrolled in the study between October 2019 and March 2020 and they were categorized into three groups: (1) patients complaining of chest pain suspected of CAD with negative Troponin I, and who underwent DSE or exercise ECG tests to “rule-in” or “rule-out” the presence of significant myocardial ischemia (*n* = 38); (2) patients presented with acute ST elevation myocardial infarction in the first 10 h from onset of symptoms with positive Troponin I or highly-sensitive Troponin T (hs-TnT) (*n* = 16); and (3) control group of healthy volunteers (*n* = 16) with normal physical examination and no history of cardiac or other system disease, who also underwent exercise ECG testing to confirm negative myocardial ischemia. The study was conducted according to the principles outlined in the Declaration of Helsinki, and approved by the Ethics Community of the Faculty of Medicine, Cairo University, Cairo, Egypt (Approval MS-62-2019). All patients provided a written, informed consent before enrollment. The study workflow is illustrated in Figure 1.

#### 4.2.2. Study Protocol

Detailed medical history was obtained from all patients submitted to the study and the presence of any of the conventional coronary artery disease risk factors was recorded, including age, diabetes mellitus, hypertension, hypercholesterolemia, smoking, previous history of coronary artery disease, myocardial infarction, percutaneous coronary intervention (PCI), and coronary artery bypass grafting (CABG). Apart from patients with acute STEMI, patients were excluded if they had active symptoms of myocardial ischemia or infarction, (within the last month), history of recent MI or PCI, positive Troponin I or T, positive C-reactive protein (CRP), history of cardiomyopathy or heart failure or congenital heart disease, end stage renal failure, hepatitis or hepatic failure, history of thoracic irradiation therapy, malignant diseases, autoimmune diseases and inflammatory diseases such as inflammatory bowel disease and arthritis, as well as contraindications to stress testing of various causes according to the European Association of Echocardiography (EAE) Expert Consensus Statement [45].

### 4.3. Dobutamine Stress Test Protocol

#### 4.3.1. Dobutamine Stress Echocardiography 

For this study, we used the European Association of Echocardiography *Expert Consensus Statement*: An iE33 (Philips Medical Systems, Andover, MA, USA) ultrasound machine [45] Studies were performed by an experienced operator. Dobutamine was intravenously infused during 3-min stages at incremental doses of 10, 20, 30, and 40 μg/kg/min until ≥85% of the age-predicted maximal heart rate was reached. ECG and symptoms were monitored continuously and blood pressure was measured every 3 min. If the target heart rate was not achieved at the peak dose of dobutamine infusion, Atropine was administrated in 0.25 mg increments to a maximal dose of 2.0 mg. Stress testing was discontinued when the target heart rate was achieved or when one of the following events occurred: (1) new wall motion abnormality in ≥2 adjacent segments, severe chest pain or dyspnea, ≥40 mmHg decrease in systolic blood pressure, severe arterial hypertension of ≥220/120 mmHg or life threatening arrhythmia [45].

#### 4.3.2. Stress ECG Protocol

The stress ECG was performed on a treadmill according to the Bruce protocol. A standard 12-lead ECG was recorded regularly prior to, during every stage level, and after the exercise rest. The test was interpreted by two expert cardiologists. An interpretation of the test included the duration of exercise, peak treadmill speed and grade, maximum heart rate and percentage of age-predicted maximal heart rate achieved “220–age (years)”, resting and peak blood pressure, functional capacity, ST-segment changes, and occurrence of arrhythmias.

Clinical findings that suggest an exercise-stress test “positive” for ischemia include exercise-induced hypotension, exercise-induced angina or anginal equivalents with significant ST-segment changes (defined by either ≥0.1 mV horizontal or down-sloping depression or ≥0.1 mV ST-segment elevation 60–80 ms after the J-point at the end of the QRS time), malignant arrhythmias, and/or appearance of an S3, S4 or heart murmur during exercise. A “negative” test result was the lack of any of the above-mentioned findings. Stress test termination criteria were defined as relevant ST-segment changes, angina pectoris, severe dyspnea and exhaustion, severe hypertension (>220 mmHg systolic), hypotension and life-threatening arrhythmias [45].

### 4.4. Gene Amplification Analysis 

Three mL blood samples were collected from patients at the presented scheduled time, as follows: (1) for STEMI patients, samples were collected immediately at presentation to hospital ED; (2) for healthy subjects, samples were collected 30 min after ECG-treadmill stress test; and (3) for the stress test group, two blood samples were collected immediately before test and 30 min after termination of dobutamine echocardiography and exercise ECG tests. To obtain the sera, the blood samples were centrifuged at 1300× *g* at 4 °C for 20 min, and the collected sera was stored at −80 °C until analyzed.

Total RNAs and miRNAs were extracted from subjects’ sera using the RNeasy Mini Kit (Qiagen, Hilden, Germany). After spectrophotometric measurement of the RNA concentration and purity, the cDNA was synthesized by reverse transcription reaction using miScript RT Kit (Qiagen, Hilden, Germany). Subsequently, the expression profiles of the five genes “*miR-137, miR-106b, FTHL-17, ANAPC11 and lnc-CTB89H12.4*” were measured using quantitative Real time PCR (qPCR). The specific primer sequence assays including *Hs_miR-137_1, Hs_miR-106b-5p_1, Hs_FTHL17_1, Hs_ANAPC11_1,* and *RT^2^_CTB89H12.4], and also Hs_RNU6-2_11* and *Hs_ GAPDH* genes, which were used as reference housekeeper gene, were purchased from (Qiagen, Hilden, Germany). Following gene amplification, the gene expression was calculated in each sample using the 2^∆∆Ct^ analysis. All PCR procedures were conducted according to the manufacture instructions. PCR was conducted on Rotor-Gene Q 5plex HRM Platform (Qiagen, Hilden, Germany). 

### 4.5. Troponin Measurement

All subjects including healthy volunteers underwent analysis of cardiac Troponin before the stress test using Elecsys Troponin I assay (Cobas e601 Analyzer, Roche Diagnostics), with the 99th percentile upper reference limit as 400 ng/L, as well as hs-Troponin T assay (Cobas 6000 Analyzer, Roche Diagnostics) with the 99th percentile upper reference limit as 5 ng/L.

### 4.6. Statistical Analysis

Statistical analysis was performed using GraphPad Prism software, (version no: 8.0.2; GraphPad Software Inc.). The median value and minimum to maximum range were used to present the skewed data, while mean ± SD were used for normally distributed numerical data, number of cases and percentage using cross-tabulation. Since, the gene expression levels are not normally distributed among groups, the comparative analysis between two groups was performed using the non-parametric Mann–Whitney U test. However, ANOVA was used to evaluate the difference in gene expression for more than two groups. The Wilcoxon signed-rank test was used to test the significant difference for gene expression between paired samples (“pre- and post-stress test”). In addition, we compared gene expression levels between the “pre-stress test” groups (negative vs positive) using the Welch’s t test. Significance was set at *p* ≤ 0.05.

## 5. Conclusions

Although Troponin helps accurate and timely diagnosis of acute (thrombotic) myocardial infarction, it is not useful in the absence of myocardial injury, even with ongoing ischemia, and it cannot confirm the diagnosis of symptomatic or silent ischemia during stress testing [30,31,32,33,34,35]. Small studies have indicated a possible incremental value of Troponin in diagnosing CAD, but larger trials are needed to verify the utility of systematic assessment in patients suspected of CAD, since biomarkers do not yet have a role in diagnosing obstructive CAD [47].

Recent gene expression testing provides a new approach for blood testing with a relatively high sensitivity and negative predictive value for CAD in symptomatic patients, but it is not widely available and has not been evaluated in asymptomatic patients [48]. Therefore, developing a simple laboratory test, like Nourin, for early identification of myocardial ischemia in individuals who have CAD in the stable state before progressing to infarction is key to providing crucial diagnostic steps to limit adverse cardiac events. Nourin-dependent *miR-137* and *miR-106b-5p* have the diagnostic potential to “rule-in” myocardial ischemia in symptomatic patients who are suspected of CAD with negative Troponin. The ability of Nourin miRNAs to diagnose myocardial ischemia in these suspected CAD patients was consistent with results obtained from DSE or exercise ECG stress tests. Furthermore, the observed elevated levels of Nourin miRNAs “pre-stress test” might be used as an early “diagnostic” test in outpatient clinics to “rule-in” patients with suspected CAD, thus providing crucial therapy to improve patients’ health outcome and save lives.

Additionally, although percutaneous coronary intervention (PCI) is widely used to treat patients with CAD, the procedure is receiving much criticism because of the limited morbidity and mortality benefits for some stable CAD patients [49]. A major limitation is the lack of a simple blood test that can verify patients with large areas of severe ischemic myocardium in the stable subset of patients with coronary artery disease, whom would likely benefit from the PCI procedure [50]. Despite our encouraging preliminary data, the small sample size is a major limitation of the current analysis and all our results should be considered as hypothesis-generating. Larger clinical studies are needed to determine whether the Nourin gene-based test: (1) can identify CAD patients with severe myocardial ischemia whom will be likely to benefit from PCI before conducting the procedure, (2) is a novel diagnostic biomarker to identify early-stage myocardial ischemia not only in symptomatic CAD patients, but also in asymptomatic, and (3) can predict severity of myocardial ischemia (mild, moderate and severe) in CAD patients.

## Figures and Tables

**Table 1:** Baseline Clinical Characteristics of Different Patient Groups.

Variable	Healthy (*n* = 16)	ECHO/ECG Stress Test		STEMI (*n* = 16)	*p*-Value
		Negative (*n* = 7)	Positive (*n* = 5)		
Age (Years) Mean ± SD	32.9 ± 9.9	53.5 ± 9.9	55 ± 4.8	54.4 ± 12.7	0.001
Sex: Males: *n* (%)	16 (100)	3 (43)	4 (80)	12 (75)	0.014
Risk Factors BMI (kg/m^2^) Mean Smoking: *n* (%) Diabetes Mellitus: *n* (%) Hypertension: *n* (%) Dyslipidemia: *n* (%)	26.8 ± 4.7 8 (50) --- --- ---	31.0 2 (29) 2 (40) 4 (57) 3 (43)	30.0 --- 5 (71) 2 (40) 2 (40)	31.4 ± 4.8 10 (52.5) 6 (37.5) 7 (43) 5 (31)	0.145 0.09 0.15 0.82 0.6

ECHO: echocardiography, ECG: electrocardiogram, STEMI: ST-segment elevation myocardial infarction, BMI: body mass index, *n*: number of cases, %: percentage of cases calculated as percentage in the group, SD: standard deviation, *p*-value > 0.05 considered statistically insignificant, *p*-value < 0.05 considered statistically significant.

**Table 2:** Post-ECHO/ECG stress test gene expression profiles of chest pain patients with positive and negative stress test versus healthy (negative controls) and STEMI (positive controls).

Variable	Negative Control	Post-ECHO/ECG Stress Test		Positive Control
	Healthy (*n* = 16) Median (min–max)	Negative (*n* = 7) Median (min–max)	Positive (*n* = 5) Median (min–max)	STEMI (*n* = 16) Median (min-max)
miR-137 (log_10_)	1.05 (0.1–3.7)	4.5 (1.5–5.0)	2575 (917–3774)	3163 (936–9878)
miR-106b (log_10_)	1.08 (0.2–3.6)	3.3 (1.8–8.5)	512 (305–803)	953 (307–2504)
FTHL-17 (log_10_)	3.8 (1.6–16.7)	1.9 (0.2–4.6)	131 (49–228)	50 (33–76)
ANAPC11 (log_10_)	0.9 (0.1–3.9)	0.2 (0.15–1.7)	11.9 (5.4–18.0)	8.4 (4.5–25.0)
CTB89H12.4 (log_10_)	65 (26–83)	0.8 (0.5–1.1)	0.07 (0.02–0.1)	0.9 (0.14–3.2)

ECHO: echocardiography, ECG: electrocardiogram, STEMI: ST-segment elevation myocardial infarction, *FTHL-17*, Ferritin heavy chain like 17, *ANAPC11*: anaphase promoting complex subunit, *CTB89H12.4* is a long non-coding RNA (lncRNA), min: minimum, and max: maximum.

## Data Availability

The data presented in this study are available on request from the corresponding author. The data presented in this study are available on request from the corresponding author. The data are not publicly due to privacy.

## Abbreviations

ACS: acute coronary syndrome, AMI: acute myocardial infarction, STEMI: ST elevation myocardial infarction, UA: unstable angina, CAD: coronary artery disease, MI: myocardial injury, CK-MB: creatine kinase isoenzyme MB, CCrP: Cyclocreatine Phosphate, ED: emergency department, ELISA: enzyme-linked immunosorbent assay, DEGs: different expressed genes, FTHL-17: Ferritin heavy chain like 17, ANAPC11: anaphase promoting complex subunit, CTB89H12.4: long coding RNA, min: minimum, max: maximum, GO: gene ontology, KEGG: Kyoto Encyclopedia of Genes and Genomes, LV: left ventricular, lncR: long non-coding RNA, miR: micro RNA, mRNA: messenger RNA, TNF-α: tumor necrosis factor-alpha, VECs: vascular endothelial cells, PCI: percutaneous coronary intervention, Isoproterenol: ISO.
